# Metal-organic cage as a theranostic nanoplatform for magnetic resonance imaging guided chemodynamic therapy

**DOI:** 10.7150/thno.97264

**Published:** 2024-08-12

**Authors:** Peilin Yin, Demei Sun, Yucen Deng, Xinyuan Zhu, Youfu Wang, Jinghui Yang, Xuesong Feng

**Affiliations:** 1 School of Pharmacy, China Medical University, Shenyang 110122, China.; 2 School of Chemistry and Chemical Engineering, Shanghai Jiao Tong University, Shanghai 200240, China.; 3 Department of Organ Transplantation, Shanghai Changzheng Hospital, Naval Medical University, Shanghai 200003, China.; 4 School of Light Industry & Chemical Engineering, Dalian Polytechnic University, Dalian 116034, China.

**Keywords:** metal organic cage, theranostics, chemodynamic therapy, magnetic resonance imaging, cervical cancer

## Abstract

**Rationale:** Theranostic nanoplatforms exert a vital role in facilitating concurrent real-time diagnosis and on-demand treatment of diseases, thereby making contributions to the improvement of therapeutic efficacy. Nevertheless, the structural intricacy and the absence of well-defined integration of dual functionality persist as challenges in the development of theranostic nanoplatforms.

**Methods:** We develop an atomically precise theranostic nanoplatform based on metal-organic cage (MOC) to provide magnetic resonance imaging (MRI) guided chemodynamic therapy (CDT) for cancer therapy and assess the theranostic performance both *in vitro* and *in vivo*. Through UV-vis spectroscopy, electron paramagnetic resonance (EPR), confocal microscopy, flow cytometry, immunofluorescence staining, and western blotting, the ability of MOC-Mn to generate •OH and the subsequent inhibition of HeLa cells was confirmed.

**Results:** The MOC-Mn composed of manganese and calixarene was successfully synthesized and comprehensively characterized. The catalytic activity of manganese within MOC-Mn facilitated the efficient generation of hydroxyl radicals (•OH) through a Fenton-like reaction, leveraging the high concentrations of hydrogen peroxide in the tumor microenvironment (TME). Additionally, its capacity to prolong the T1 relaxation time and augment the MR signal was observed. The theranostic efficacy was verified *via* rigorous* in vitro* and *in vivo* experiments, indicating that MOC-Mn offered clearer visualization of tumor particulars and substantial suppression of tumor growth.

**Conclusion:** This study showcases a precise MRI-guided CDT theranostic nanoplatform for cancer therapy, thereby promoting the advancement of precise nanomedicine and structure-function research.

## Introduction

Theranostic nanoplatforms integrating diagnostic and therapeutic functions have garnered significant attention due to their ability to provide real-time monitoring during treatment and adjust the treatment based on feedback 1-3. However, the technical complexity and ambiguous structures of developed theranostic nanoplatforms leading to uncertain biocompatibility and poor quality control are challenges that need to be addressed for effective clinical application 4. Specifically, theranostic nanoplatforms based on MRI technology have attracted interest because of their inherent advantages such as non-invasiveness, high spatial resolution, and minimal tissue damage 5-7. By utilizing the principle of nuclear magnetic resonance, these platforms can visualize the internal structure of the human body by detecting and analyzing signals from hydrogen nuclei within the body 8. Paramagnetic metallic elements (Mn, Gd, Fe) are commonly used as longitudinal (T1) or transverse (T2) contrast agents 9, however, their construction is often complex and their compositions may be toxic 10. In addition, as an emerging anticancer therapeutic strategy, CDT can effectively inhibit cancer cell proliferation by converting endogenous H_2_O_2_ in the tumor microenvironment into toxic •OH through Fenton or Fenton-like reactions 11-14. Thus, there is a pressing clinical need to develop precise MRI-guided CDT theranostic nanoplatforms with clarified structure-function relationships and promising prospects for clinical application.

Metal-organic cages have emerged as a prominent candidate among precise nanostructures due to their unique combination of organic ligands and metal clusters or ions 15, 16. MOCs exhibit precise, discrete, and customizable nanoarchitectures at the atomic level, along with independent cavities 17, 18. The structure and performance of MOCs can be tailored by adjusting the selection and stoichiometry of metal clusters or ions to organic ligands, thereby significantly expanding the diversity of MOCs 19, 20. With their versatility, ultra-small size, facile functionalization and high stability, MOCs offer new opportunities for addressing key challenges in fields such as energy storage 21, 22, photovoltaics 23, catalysis 24, and nanomedicine 25. In fact, MOCs have found extensive applications in the field of medicine, including cancer therapy 26-28, imaging and sensing technologies 29-34, anti-inflammatory treatments 35, and antibacterial interventions 36-38.

**Scheme 1:**
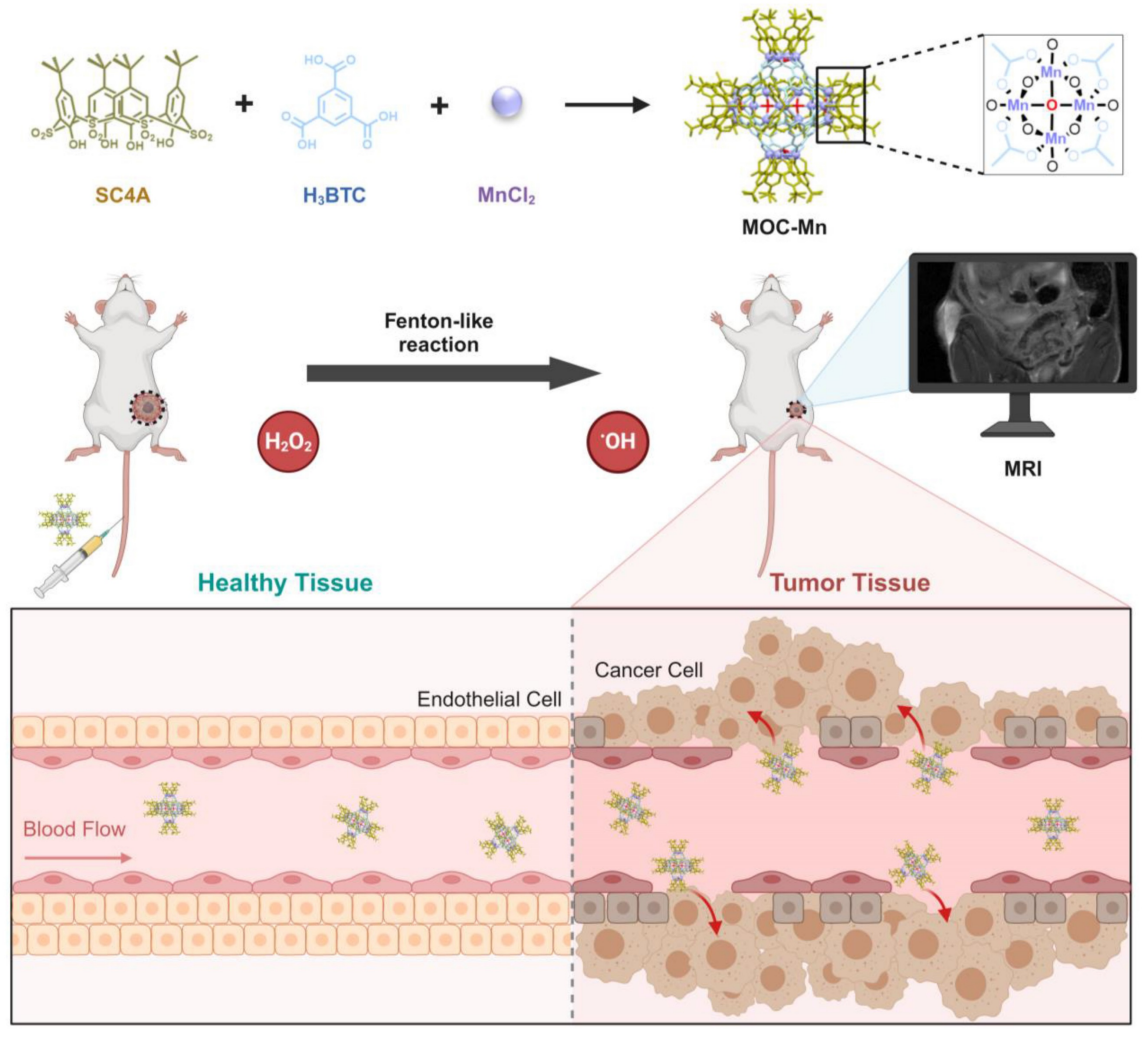
The synthetic pathway of MOC-Mn and its application in MRI-guided CDT for anti-tumor therapy. This figure is created with BioRender.com.

To develop an atomically precise theranostic nanoplatform based on MOC, we selected Mn as the active centers in MOC due to its outstanding MRI functionality and Fenton-like property. Additionally, Mn-based MOCs are known for their high stability and biocompatibility, attributed to the difficulty of Mn leakage 39, 40. Herein, we present calixarene-blocked MOC-Mn as a theranostic nano-agent for T1-weighted MRI-guided chemodynamic therapy (Scheme 1). Through a solvothermal method, Mn ions can coordinate with 4-tert-butylsulfonylcalix[4]arene (SC4A) and 1,3,5-benzenetricarboxylic acid (H_3_BTC) to form octahedral MOC-Mn which further stack to MOC crystal. The ability of MOC-Mn to generate reactive oxygen species (ROS) and prolong the relaxation signal was demonstrated through *in vitro* experiments. Furthermore, *in vivo* experiments confirmed the excellent biocompatibility and theranostic performance of MOC-Mn, as evidenced by improved visualization of tumor details and significant inhibition of tumor growth.

## Methods

### Materials

All chemicals and solvents are commercially available and used without further purification. MnCl_2·_4H_2_O, methylene blue (MB), potassium dihydrogen phosphate (KH_2_PO_4_), potassium hydrogen phosphate (K_2_HPO_4_), sodium acetate (NaAc), sodium hydroxide (NaOH), sodium perborate tetrahydrate (NaBO_3_·4H_2_O), N,N-dimethylformamide (DMF), methanol (MeOH), glacial acetic acid (HAc), and dimethylsulfoxide (DMSO) were purchased from Shanghai Titan Scientific Co., Ltd. (China). Benzene-1,3,5-tricarboxylic acid (H_3_BTC), *p*-tertbutylphenol, 3,3',5,5'-tetramethylbenzidine (TMB), and tetraethylene glycol dimethyl ether were purchased from Bide Pharmatech Co., Ltd. (China). Chloroform (CHCl_3_), diethyl ether, H_2_O_2_, sulfur (S), H_2_SO_4_, HCl, and HNO_3_ were purchased from Sinopharm Chemical Reagent Co., Ltd. (China). 4-tert-Butylsulfonylcalix[4]arene (SC4A) was synthesized according to previous reports. MTT cell proliferation and cytotoxicity assay kit (C0009S), reactive oxygen species detection kit (S0033S), mitochondrial membrane potential assay kit with JC-1, and all materials of western blot (WB) were purchased from Beyotime Biotech. Inc. Saline, paraformaldehyde, and cephaloquinoxime for animal use were purchased from Beyotime Biotech. Inc. HeLa cells, fetal bovine serum (FBS), and Dulbecco's modified Eagle medium (DMEM) were purchased from Beyotime Biotech. Inc. Isoflurane was purchased from Rayward Life Technology Co., Ltd. (Shenzhen, China) The deionized water was used in the preparation of aqueous solutions (18.25 MΩ).

### Characterizations

Powder X-ray diffraction (PXRD) spectra were measured on a D8 ADVANCE Da Vinci diffractometer using Cu Kα radiation. Morphology images and energy dispersive spectrum (EDS) analysis were recorded on a Apreo 2S field emission scanning electron microscope (SEM) at 15 kV acceleration voltage. Mass spectrum was measured on a Bruker matrix assisted laser desorption/ionization time of flight (MALDI-TOF) mass spectrometry. X-ray photoelectron spectroscopy (XPS) was analyzed using a ESXCALAB Xi+ instrument from Thermo Scientific and the C1s peak at 284.8 eV. UV-vis spectra were recorded on a UV-2600i (Shimadzu). Flourier transform infrared spectroscopy (FT-IR) was performed on a Nicolet iS50 using an attenuated total refraction (ATR) attachment. Particle Size and Zeta Potential Analyzer (ZS90) was employed to test the particle size distribution and zeta potential through dynamic light scattering (DLS). The crystal structure was determined on single-crystal X-ray diffractometer (D8 Venture). The generation of free radicals was assessed using electron paramagnetic resonance (EPR) spectroscopy (Bruker EMXplus). Inductively coupled plasma mass spectrometry (ICP-MS) (i CAP Q, Thermo) were used to detect cellular uptake.

### Synthesis of MOC-Mn

MnCl_2_·4H_2_O (0.05 mM), SC4A (0.01 mM) 41, and H_3_BTC (0.03 mM) were combined in N,N-dimethylformamide (1 mL) and methanol (20 μL) and heated at 100 °C for 24 h. Upon cooling, large purple crystals with cubic morphology were obtained through centrifugation and subsequent drying.

### Detection of •OH by TMB

The generation of •OH was monitored by measuring the absorbance change at 652 nm, which corresponds to the characteristic peak of TMB 42. In this investigation, MOC-Mn (2 mg/mL, 50 μL), TMB (12 mg/mL, 50 μL), and H_2_O_2_ (0.4 M, 50 μL) were co-incubated in acetate buffer (pH = 3.6, 2 mL). Spectra were recorded every 10 min at room temperature. Furthermore, the impact of different reaction substrates on the catalyzed reaction was also explored through four groups: (1) TMB+H_2_O_2_; (2) MOC-Mn+H_2_O_2_; (3) MOC-Mn+TMB; (4) MOC-Mn+TMB+H_2_O_2_.

### Detection of •OH by MB

The absorbance variation at the characteristic peak of MB (650 nm) was directly correlated with the level of •OH generation 43. In this study, a co-incubation of MOC-Mn (2 mg/mL, 50 μL), MB (1 mg/mL, 50 μL), and H_2_O_2_ (0.4 M, 50 μL) in phosphate buffer (pH = 6.5 or 7.4, 2 mL) was conducted and the spectra were recorded every 10 min at room temperature. The effect of different reaction substrates on the catalyzed reaction was also investigated through four control groups: (1) MB+H_2_O_2_; (2) MOC-Mn+H_2_O_2_; (3) MOC-Mn+MB; (4) MOC-Mn+MB+H_2_O_2_.

### Detection of •OH by EPR

•OH was identified and quantified using EPR spectra due to its radical nature 44. In this study, DMPO was employed as a trapping agent to investigate the influence of different time intervals (0 or 5 min) and pH levels (pH = 6.5 or 7.4) on the generation of •OH by MOC-Mn.

### *In vitro* MRI of MOC-Mn

The concentrations of Mn^2+^ in MnCl_2_ and MOC-Mn were standardized (0 mM, 0.1 mM, 0.2 mM, 0.4 mM, and 0.6 mM) for comparative assessment of their imaging performance 45. Subsequently, these solutions were transferred into 1.5 mL EP tubes for MR image acquisition and relaxation time measurements using a 0.5 T MR imager (MesoMR23-060H-I) at room temperature.

### Hemolytic assessment

The fresh whole blood of mice was collected in 2 mL EP tubes and centrifuged at 3000 rpm for 5 min to separate the upper serum from the red blood cells (RBCs). The RBCs were then washed three times with PBS and diluted to achieve a concentration of a 5% RBC suspension. Following this step, MOC-Mn was dissolved in DMSO at a concentration of 2 mg/mL and subsequently added to PBS to achieve final concentrations of 25, 50, 75, 100, and 125 μg/mL. PBS (1 mL) and double distilled water (DDW, 1 mL) served as negative and positive controls, respectively. The samples underwent thorough mixing before being incubated for 4 h prior to observation.

### *In vitro* cytotoxicity experiment

HeLa cells and human kidney 2 (HK-2) cells were cultured in DMEM medium supplemented with 10% fetal bovine serum, and 1% penicillin-streptomycin solution. The cells were maintained at 37 °C in a humidified incubator under a 5% CO_2_ atmosphere. The cytotoxic effects of MOC-Mn on HeLa cells and HK-2 cells were evaluated using the methyl thiazolyl tetrazolium (MTT) assay. Briefly, 7000 HK-2 and HeLa cells were seeded in 96-well plates and incubated at 37 °C for 12 h. MOC-Mn was dissolved in DMSO at a concentration of 2 mg/mL and subsequently added to PBS to achieve final concentrations of 25, 50, 75, 100, and 125 μg/mL. The medium was then replaced with fresh medium containing different concentrations of MOC-Mn for an additional 24 h of incubation. Subsequently, the cells were washed three times with PBS before adding MTT working solution and incubating at 37 °C for another 4 h. After removing the MTT solution, Formazan was added and incubated on a shaker for 8 min to ensure complete dissolution. The absorbance (OD) at a wavelength of 570 nm was measured using a microreader.

### *In vitro* cellular uptake and retention

The HeLa cells were seeded into 96-well plates at a density of 10,000 cells/mL and incubated for 24 h. Subsequently, MOC-Mn was dissolved in DMSO at a concentration of 2 mg/mL and subsequently added to PBS to achieve final concentrations of 125 μg/mL (0.015 mM). MOC-Mn was then added to each plate, and the plates were subjected to culturing for 0, 4, 8, 12, 12+4, and 12+8 h. The groups labeled as "12+4 h" and "12+8 h" refer to the cells co-cultured with MOC-Mn for 12 h followed by a medium change and an additional incubation of 4 or 8 h. After digestion in trypsin-EDTA solution, the treated cells were collected by centrifugation and washed twice with PBS. The cell samples were digested in a solution containing HNO_3_ (68%) and HCl (38%) at a temperature of 100 °C for 4 h. Following cooling to room temperature, the sample was diluted to a volume of 10 mL with 2% HCl for ICP-MS analysis 46.

### *In vitro* ROS determination

Cell-permeable fluorescent and chemiluminescent probes are commonly employed for the evaluation of cellular free radical levels. In this study, we utilized 2',7'-dichlorodihydrofluorescein diacetate (DCFH-DA) to quantify the •OH levels in cells. DCFH-DA itself does not exhibit fluorescence but is capable of freely traversing the cell membrane. Upon entry into the cell, it undergoes hydrolysis by intracellular esterase to generate DCFH. DCFH is unable to permeate the cell membrane, facilitating easy loading of the probe into the cell. Intracellular ROS can oxidize non-fluorescent DCFH into fluorescent 2',7'-dichlorofluorescein (DCF). The fluorescence intensity of DCF can be used to determine intracellular ROS levels.

A commercial ROS assay kit based on DCFH-DA (Beyotime, China) was employed to quantify the level of •OH in HeLa cells. The cells were seeded into individual wells of 96-well plates and allowed to culture overnight. Then, the media was replaced with serum-free DMEM. Subsequently, MnCl_2_ (125 μg/mL, 0.63 mM) or MOC-Mn (125 μg/mL, 0.015 mM) was co-cultured with the HeLa cells for 6 h. Following incubation, the cells were detached using trypsin, washed twice with PBS, and then stained with DCFH-DA according to the provided protocol. After a 20 min incubation period, the cells were washed three times with serum-free medium and all samples were analyzed by flow cytometry (BD Biosciences, Franklin Lakes, NJ, USA) within 1 h. Additionally, confocal microscopy was utilized to detect the fluorescence of DCF. Furthermore, Hoechst 33342 staining was performed on HeLa cells for 5 min followed by observation of cell fluorescence using confocal microscopy (Nikon A1Si).

### JC-1 staining

The cells were seeded into individual wells of 96-well plates and incubated overnight. Then, the media was replaced with serum-free DMEM. Subsequently, MnCl_2_ (125 μg/mL, 0.63 mM) or MOC-Mn (125 μg/mL, 0.015 mM) was co-cultured with the HeLa cells for 6 h. After that, cells were collected and labeled with fluorescent probe JC-1 kit (Beyotime) according to the manual. The fluorescence intensity of the JC-1 and its localization were observed using flow cytometry or confocal laser scanning microscope.

### Animals and tumor models

Male nude mice (4-6 weeks old) were procured from Phenotek Biotechnology (Shanghai, China). All animal experiments were conducted in compliance with the National Institute of Health Guidelines for the Care and Use of Laboratory Animals and approved by the Scientific Investigation Board of Shanghai Changzheng Hospital (No. 202403010A). HeLa cells (2×10^6^, 100 μL) were subcutaneously injected under the right armpit to establish xenograft tumor models. The mice were randomly allocated into three groups (n = 5): Control (vehicle), MnCl_2_, and MOC-Mn when the tumors reached a size of approximately 100 mm^3^. For *in vivo* treatment, MOC-Mn was dissolved in DMSO at a concentration of 2 mg/mL and subsequently added to PBS to achieve final concentrations of 125 μg/mL (0.015 mM).

### *In vivo* MR imaging

The mice were anesthetized with isoflurane and underwent MR imaging on a 0.5 T MR scanner under the following conditions: a repetition time of 400 ms, an echo time of 18.2 ms, a slice width of 2.5 mm, and a gap of 1.0 mm.

### *In vivo* antitumor efficacy

Three groups were administered with vehicle, 100 μL of MnCl_2_ (125 μg/mL, 0.63 mM), and 100 μL of MOC-Mn (125 μg/mL, 0.015 mM) every 2 days. Body weights were measured using a weighing scale every 2 days. Tumor volumes (V) were calculated using the equation 0.52×L(length)×W^2^(width), then normalized to their initial volume (V_0_) to obtain the relative tumor volume (V/V_0_).

### Histopathological evaluation

The mice were sacrificed 2 weeks following the initiation of the treatment. The mouse tumors were sectioned coronally, fixed in 10% buffered formalin, embedded in paraffin, and cut into 3 μm thick sections. These sections were then stained with hematoxylin and eosin for histological evaluation.

### Immunofluorescence assay

The frozen sections of tumor were processed following a standardized protocol. Subsequently, the sections were incubated overnight with primary antibody against DHE (Beyotime). Following this step, the slides underwent three washes with PBS and were then incubated with secondary antibodies (Beyotime) at room temperature for 1 h. Nuclei staining was performed using DAPI (Beyotime). Finally, the slides were examined and imaged under a fluorescent microscope (Nikon 80i, Tochigi, Japan).

### Western blotting

Cells were rinsed with PBS twice and lysed in ice-cold RIPA buffer (Roche, Basel, Switzerland) containing phosphatase and protease inhibitors. Sample proteins were then subjected to 12% sodium dodecyl sulfate-polyacrylamide gel electrophoresis and transferred to nitrocellulose membranes. Membranes were probed with NLRP3, cleaved caspase-1, pro-IL-1β, and caspase-11 antibodies from Beyotime Biotech. The relative quantity of proteins was determined by a densitometer software (ImageJ, NIH, USA).

## Results

### Synthesis and characterization of MOC-Mn

**Figure 1:**
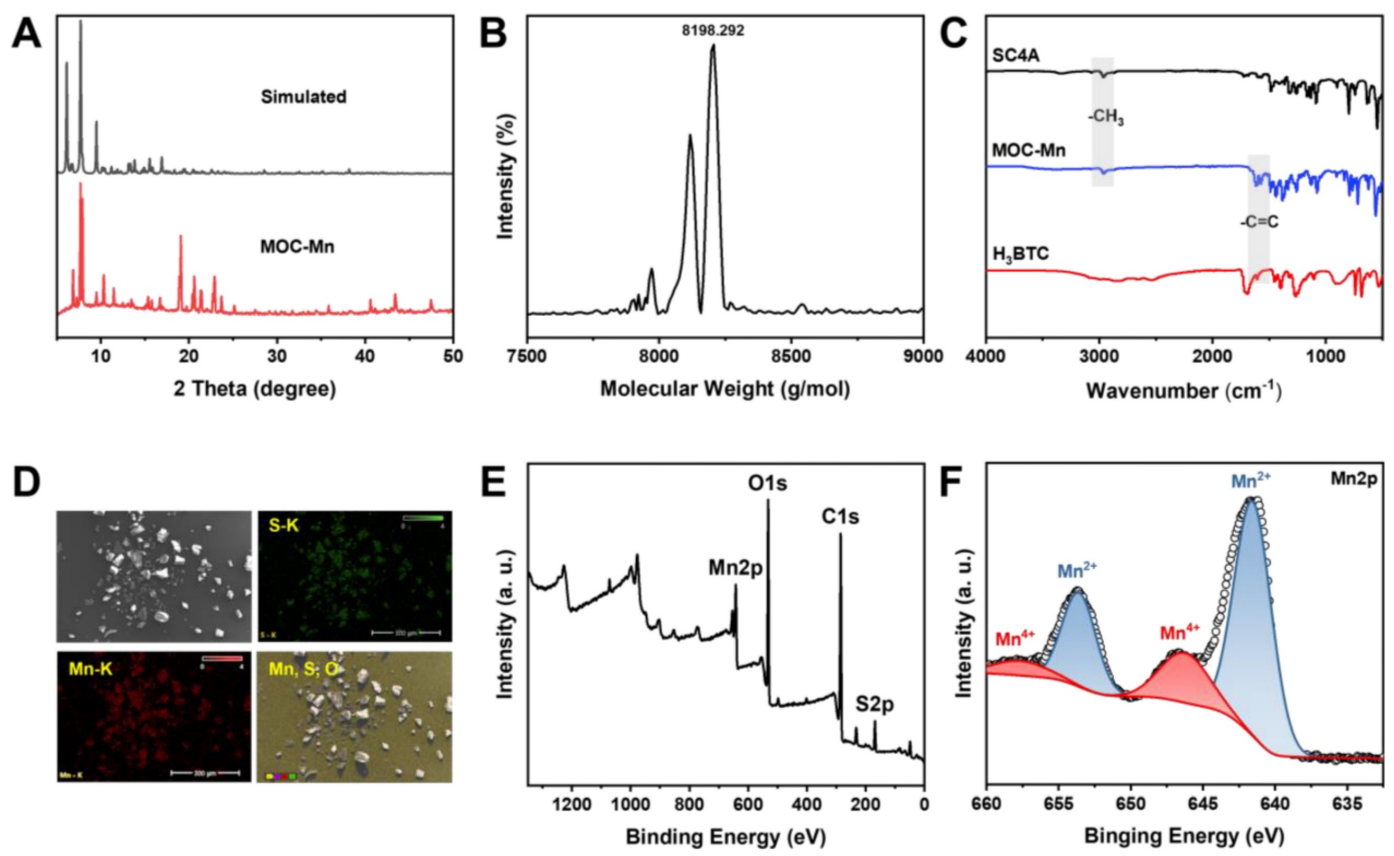
Structural characterizations of MOC-Mn. (A) PXRD and simulated pattern of MOC-Mn. (B) MALDI-TOF mass spectrum of MOC-Mn. (C) FT-IR spectra of SC4A, H_3_BTC, and MOC-Mn. (D) SEM image and corresponding element mapping of MOC-Mn. (E) XPS spectra of MOC-Mn. (F) XPS high resolution spectrum of Mn 2p.

The Crystal MOC-Mn was synthesized from MnCl_2_, H_3_BTC, and SC4A using the solvothermal method and examined under an optical microscope (Figure S1). To confirm the successful synthesis, PXRD analysis was conducted on the obtained crystals after air drying (Figure 1A). The diffraction peaks closely matched with the single crystal simulation data, providing evidence for the precise configuration of octahedral MOC-Mn with a formula of [(Mn_4_O)_6_(SC4A)_6_(BTC)_8_]. The results of single-crystal X-ray diffraction (SC-XRD) further demonstrated that the octahedral coordination MOC-Mn crystallized within the central-symmetric tetragonal space group *I*4m. There exist four crystallographically independent Mn sites, each being six-coordinated by two phenoxy μ_2_-O atoms, one axial sulfonyl oxygen atom, one μ_4_-oxygen, and two carboxyl oxygen atoms from distinct H_3_BTC ligands (Table S1 and Figure S2). The measured molecular weight of MOC-Mn from MALDI-TOF mass spectroscopy was 8198.292 Da, roughly consistent with the theoretical value of 8141.14 (Figure 1B). The higher measured value may come from the encapsulated solvents within the cavity. From FT-IR spectroscopy shown in Figure 1C, we observed characteristic peaks of MOC-Mn at 1600 and 2957 cm^-1^ from H_3_BTC and SC4A, respectively, indicating the integration of related building blocks. The morphology and elemental distributions of crystal MOC-Mn were observed using SEM and energy dispersive spectrometer. The amorphous morphology observed in the SEM emerged as a consequence of damage induced during the sample preparation process. From the outcomes of SEM and microscopic imaging, it becomes evident that the crystal we obtained was tetragonal, and the size of the crystal is approximately 100 μm. The polyhedral morphology with sharp edges demonstrated high crystallinity. The uniform distribution of Mn and S within the crystal MOC-Mn also manifested the molecular combination of these blocks (Figure 1D). The elemental analysis results were basically consistent with the theoretical composition (Table S2). XPS further confirmed the presence of C, O, S, Mn within MOC-Mn (Figure 1E). A high-resolution analysis of Mn 2p was conducted to investigate its bonding environment and states (Figure 1F). The observed four peaks were assigned to Mn^2+^ at 642.03 and 653.93 eV, and Mn^4+^ at 646.18 and 657.73 eV in two spin orbitals of Mn 2p_3/2_ and Mn 2p_1/2_, respectively. Given that Mn^2+^ was used as the source in the construction of MOC-Mn, the emergence of newly appeared Mn^4+^ during the assembly indicated its potential for redox processes. Since a crystal is composed of multiple MOCs, the particle size of MOC-Mn was examined in PBS. MOC-Mn exhibits a hydrated particle size of roughly 7.6 nm, along with narrow polydispersity indices (PDIs) of 0.15 (Figure S3). The surface charge of MOC-Mn was also determined to be weakly negative at approximately -3.12 eV (Figure S4). To facilitate subsequent tests, we conducted a preliminary assessment of the solubility and stability of MOC-Mn. It was revealed that MOC-Mn dissolved in DMSO well, and the resulting DMSO solution of MOC-Mn exhibited excellent dispersion and dispersion stability in PBS or fetal bovine serum (FBS) (Figure S3 and S5-S9).

### Detection of generated •OH

**Figure 2:**
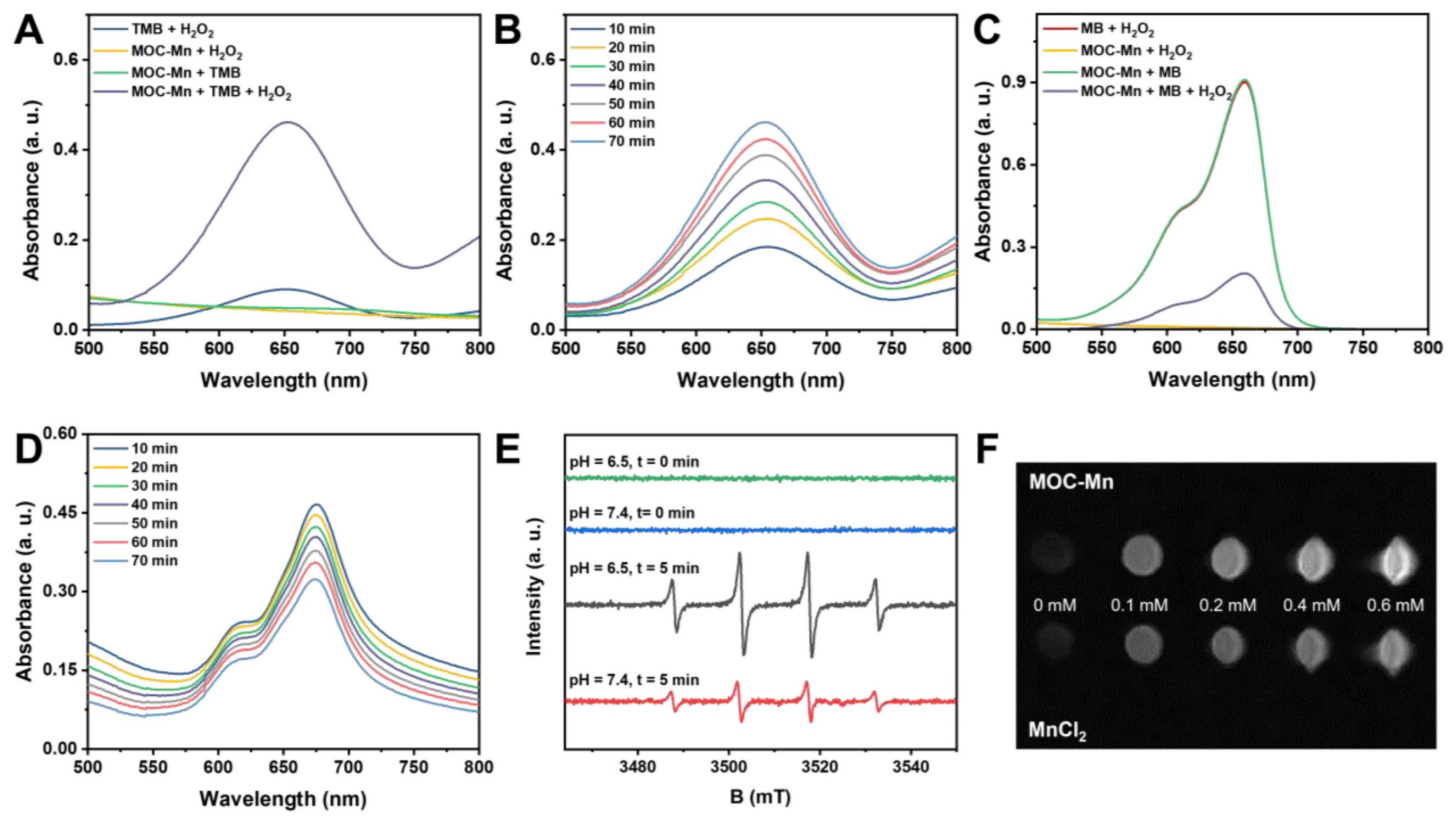
Fenton-like activity of MOC-Mn. UV-vis spectra of (A) TMB and (C) MB after different treatments. The UV-vis absorption spectra of (B) TMB and (D) MB after treatment with MOC-Mn at different time points. (E) •OH detection by EPR signals under different conditions. (F) T1-weighted images at different concentrations of Mn ion.

After successfully synthesizing and demonstrating the potential redox activity of MOC-Mn, we evaluated its ability to convert H_2_O_2_ into more potent ROS through a Fenton-like reaction. The spectral method is a commonly used strategy for detecting the decomposition of H_2_O_2_ and the generation of •OH using TMB or MB as indicators. Control experiments were initially conducted to confirm that changes in absorbance values were not simply due to the substrate itself (Figure 2A-D). In the case of TMB, the oxidized product generated during this process exhibited maximum absorbance at 652 nm, and we traced the catalytic activity of MOC-Mn towards H_2_O_2_ in solutions at different time intervals. The characteristic absorption peaks showed time-dependent enhancement, as indicated by the gradual blue coloration of the reaction solution. For MB, it can be reduced by generated •OH, leading to a transition from its blue color to a colorless state. Therefore, the generation of •OH was assessed by observing time-dependent spectral decreases in MB at 660 nm. Our results demonstrated that MOC-Mn is indeed capable of catalyzing H_2_O_2_ to produce •OH.

EPR is a spectroscopic technique used to detect free radicals based on their spectral characteristics. 5,5-dimethyl-1-pyrroline N-oxide (DMPO) was chosen as a trapping agent for hydroxyl radicals (•OH). The EPR spectrum displays characteristic peaks of •OH, with four peaks appearing in a 1:2:2:1 ratio from left to right (Figure 2E). We also investigated the impact of pH on •OH generation and found that the signal of •OH generated by MOC-Mn was weaker under neutral conditions compared to acidic conditions, indicating that MOC-Mn exhibited greater capacity for •OH generation in acidic environments (Figure S10). These findings were consistent with the spectral results, demonstrating the ability of MOC-Mn to catalyze H_2_O_2_ production of •OH.

### *In vitro* MRI of MOC-Mn

The ability of MRI to provide high-quality, non-invasive images of tissues and organs has revolutionized modern medicine, enabled more accurate diagnoses and effective treatments while reduced the risk of complications associated with invasive procedures. Previous studies have demonstrated that paramagnetic transition metals can effectively enhance the relaxation effect, but their cardiotoxicity limits their application 47. Therefore, MOC-Mn stabilizes Mn ions in the skeleton to mitigate toxicity while ensuring optimal imaging results. The imaging capability and longitudinal relaxation rate (r1) of MnCl_2_ and MOC-Mn in aqueous solution was quantified using a 0.5T MRI scanner (Figure 2F and Figure S11). An increase in concentration resulted in stronger contrast and enhanced relaxation observed in the T1 weighted MR signal intensity of both MOC-Mn and MnCl_2_, indicating a concentration-dependent performance. MOC-Mn exhibited an r1 value of 7.02 mM^-1^S^-1^, while the r1 value of MnCl_2_ was calculated to be 8.93 mM^-1^S^-1^. These findings highlight the potential of MOC-Mn as a promising T1 contrast agent for diagnosis.

### Cellular uptake and biocompatibility of MOC-Mn

**Figure 3:**
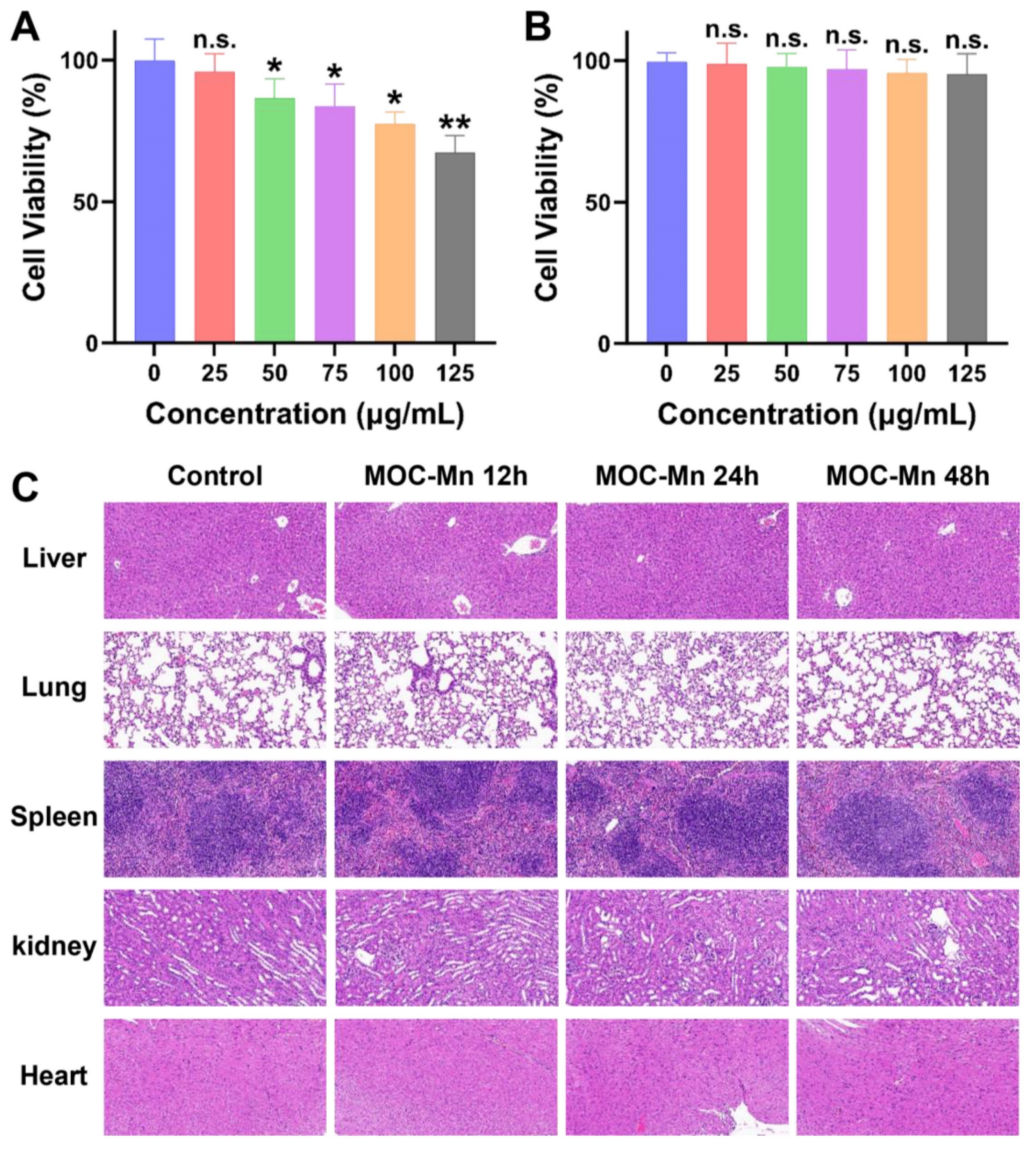
Cytotoxicity and biocompatibility of MOC-Mn. MTT assay of (A) HeLa cells and (B) HK-2 cells. (C) Histological examination using H&E staining (20×) performed on the major organs of the mice following treatment with MOC-Mn. Data are presented as mean ± s.d. n.s. *p* > 0.05, * *p* < 0.05, ** *p* < 0.01.

To further advance our biological research, we conducted a comprehensive investigation into the cellular uptake of MOC-Mn and its toxicity both *in vitro* and *in vivo*. The hemolytic activity of MOC-Mn against erythrocytes was firstly studied (Figure S12). Within the concentration range of 0-125 μg/mL, no evident erythrocyte rupture was observed in the MOC-Mn groups, presenting a stark contrast to the group treated with DDW. In Figure 3A-B, we simultaneously assessed the viability of both normal cells (HK-2) and tumor cells (HeLa) exposed to MOC-Mn. For HeLa cells, there was a gradual decline in cell viability with increasing concentrations of MOC-Mn. Conversely, HK-2 cells exhibited a survival rate of 90% or higher within the concentration range of 0-125 μg/mL, suggesting that MOC-Mn did not demonstrate cytotoxicity towards normal cells.

The effective uptake and utilization of medical agents are essential for pharmacological efficacy. Therefore, we investigated the uptake of MOC-Mn (125 μg/mL, 0.015 mM) by HeLa cells after the treatment and its subsequent retention effect (Figure S13). The results showed a steady increase in Mn content in the cells over this time period. Even after removing the culture medium with MOC-Mn at 4 and 8 h, the measured Mn content only exhibited a slight decrease compared to that at 12 h, indicating high *in vitro* uptake rate and excellent retention of MOC-Mn. Furthermore, H&E staining of sections from major organs (heart, lung, spleen, kidney, and liver) revealed no significant tissue damage after treatment with MOC-Mn for 12-48 h, demonstrating excellent biocompatibility at an administered dose of 125 μg/mL (0.015 mM) (Figure 3C).

### Evaluation of intracellular •OH

**Figure 4:**
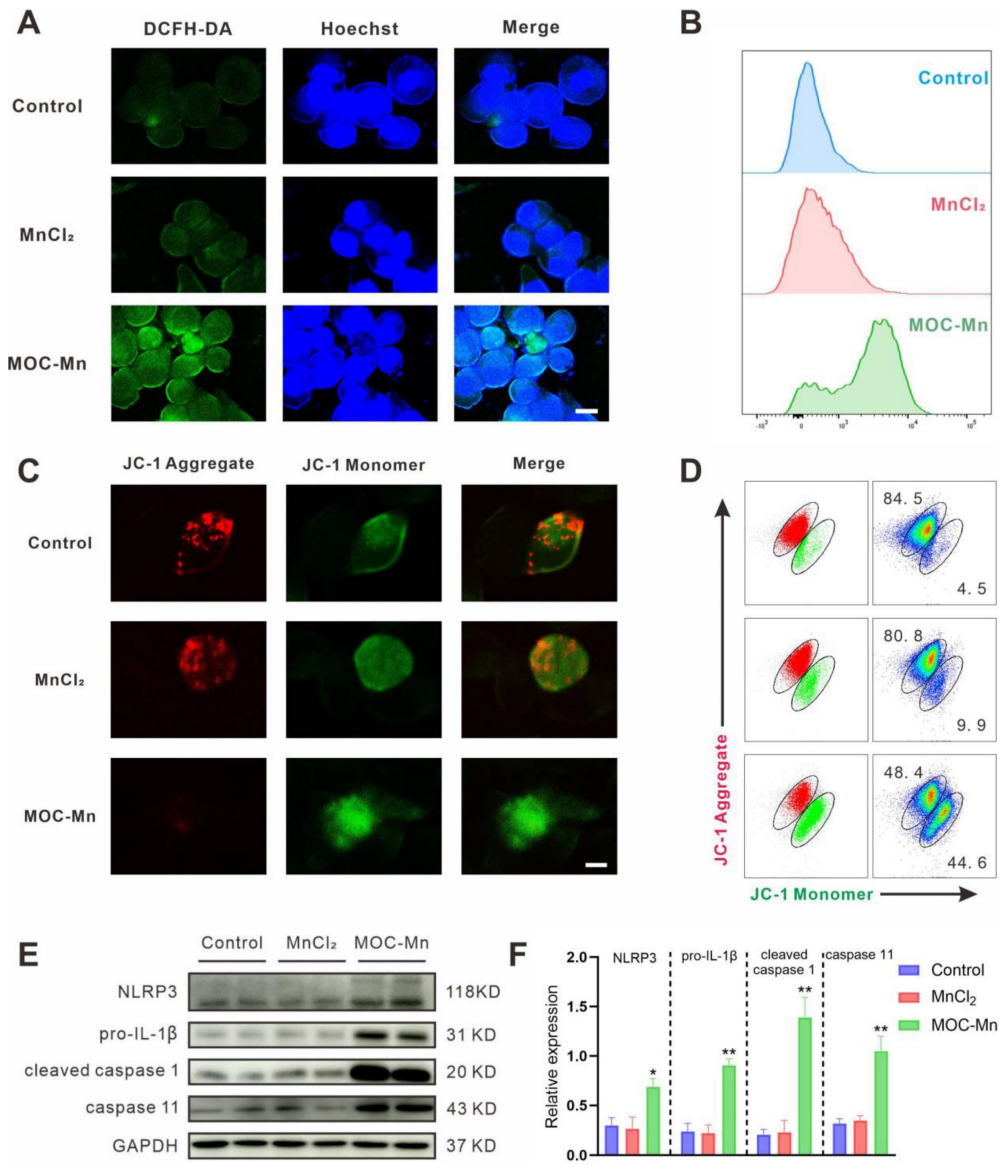
Assessment of intracellular ROS levels. (A) Visualization of ROS in HeLa cells using confocal microscopy. Blue fluorescence indicates the stained cellular nucleus with Hoechst 33342, while green fluorescence represents the stained ROS with DCFH-DA. Scale bars represent 5 μm. (B) Flow cytometry histogram depicting the results of HeLa cells treated with PBS, MnCl_2_ and MOC-Mn. (C) Representative fluorescence images of HeLa stained with JC-1. Scale bars represent 5 μm. (D) Analysis of mitochondrial membrane potential of HeLa cells after MOC-Mn treatment using JC-1 staining. (E) Western-blot identification of pyroptosis-associated proteins including NLRP3, pro-IL-1β, cleaved caspase 1, and caspase 11. (F) The relative densities of the bands in each lane were analyzed and normalized to GAPDH. Data are presented as mean ± s.d. * *p* < 0.05, ** *p* < 0.01.

In order to investigate the mechanism of carcinoma inhibition and confirm the Fenton-like activity at the cellular level, we quantified intracellular •OH levels using flow cytometry, confocal microscopy, and fluorescence staining techniques. Intracellular ROS can oxidize non-fluorescent DCFH to yield fluorescent DCF, making DCFH-DA a reliable indicator for monitoring ROS levels. In confocal microscopy analysis, the homogeneous and intense green fluorescence in cells treated with MOC-Mn was observed, suggesting a substantial increase in ROS levels in HeLa cells (Figure 4A). This finding was further confirmed by flow cytometry, which indicated a significant rise in fluorescent intensity following MOC-Mn administration (Figure 4B). Additionally, as free radical damage can lead to mitochondrial dysfunction in cells, we further explored the depolarizing effect of mitochondrial membranes in HeLa cells using dual fluorescent JC-1 (Figure 4C-D). The results indicated that MOC-Mn treatment significantly decreased the ratio of JC-1 aggregate (red fluorescence signal) to JC-1 monomer (green fluorescence signal), suggesting a reduced mitochondrial membrane potential due to accumulated ROS.

Given that updated molecular insights have elucidated the significance of mitochondria in pyroptosis and its implications in various cancers 48, 49, we therefore investigated whether the inhibitory effect of MOC-Mn on HeLa cells is through mitochondrial depolarization and subsequent induction of pyroptosis. As expected, MOC-Mn treatment markedly increased the expression levels of pyroptosis activation-associated proteins NLRP3, pro-IL-1β, cleaved caspase 1, and caspase 11 (Figure 4E-F).

### Anticancer and MRI efficacy of MOC-Mn

**Figure 5:**
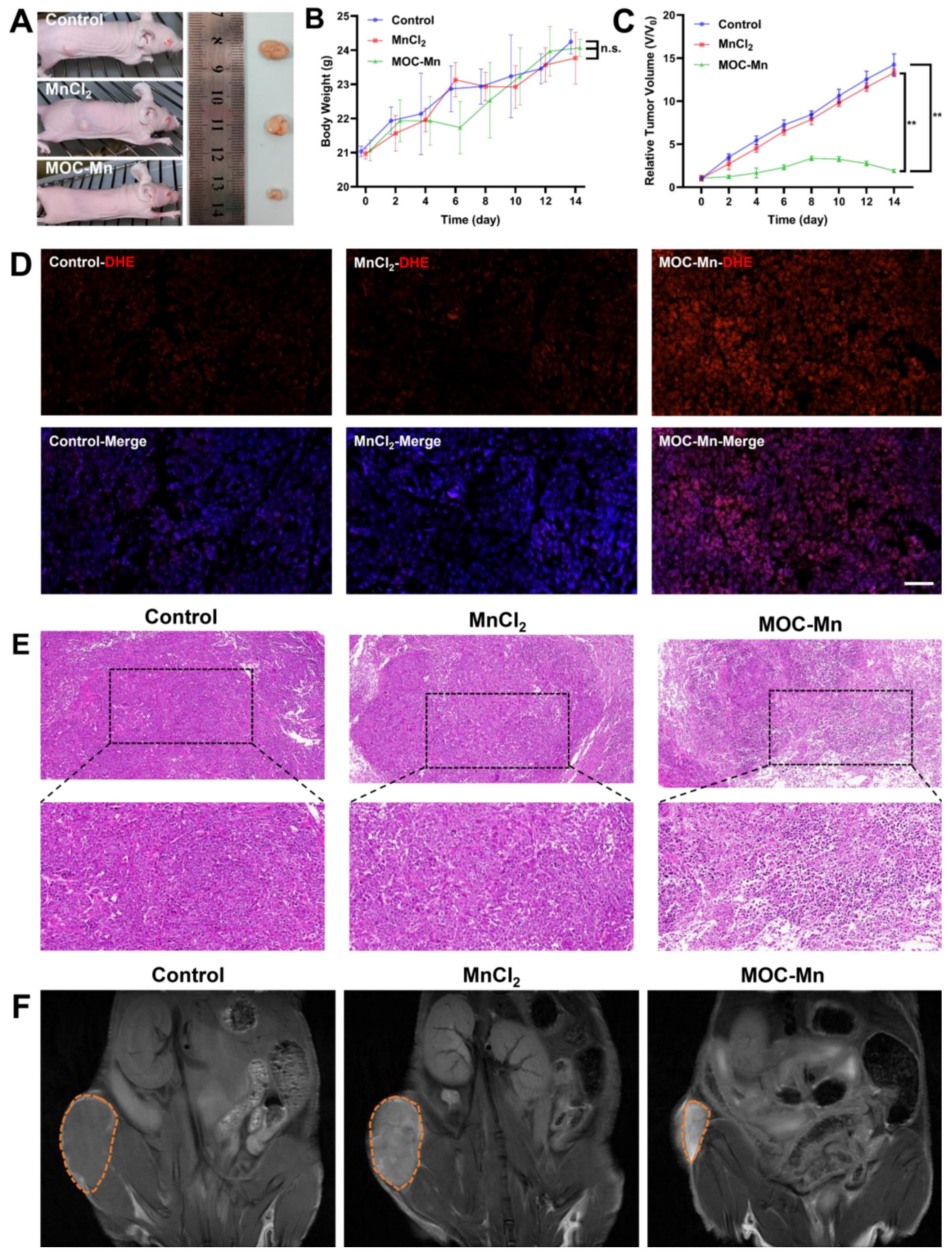
The* in vivo* theranostic performance of MOC-Mn. (A) Representative tumor-bearing mice and isolated tumor tissue under different treatments. (B) Changes in the body weight of mice during treatment. (C) Tumor volume in different treatment groups. (D) Representative immunofluorescence images of DHE expression. Scale bars represent 50 μm. (E) H&E staining (20×, 40×) performed on tumors from different groups. (F) *In vivo* T1 MR images of different groups. Data are presented as mean ± s.d. n.s. *p* > 0.05, ** *p* < 0.01.

Given the satisfactory biocompatibility, •OH generation ability, and observed *in vitro* MRI effect of MOC-Mn, further investigations were undertaken to assess its *in vivo* anti-tumour efficacy and MRI capabilities. Mice were classified into three groups and were administered with vehicle, 100 μL of MnCl_2_ (125 μg/mL, 0.63 mM), and 100 μL of MOC-Mn (125 μg/mL, 0.015 mM) every two days. The representative tumour growth conditions in different groups were illustrated in Figure 5A. The MOC-Mn group exhibited superior tumour inhibitory activity compared to the other two groups. Throughout the treatment period, we monitored changes in mouse body weight and observed no significant weight loss, thus confirming the low toxicity of MOC-Mn (Figure 5B). It should be noted that fluctuations in body weight may also be influenced by tumour size. Compared with baseline measurements, the tumor volume increased by 14.2-fold for mice treated with vehicle, 13.2-fold for those treated with MnCl_2_, and only 1.9-fold for those treated with MOC-Mn (Figure 5C). We used DHE as an intracellular superoxide indicator which undergoes oxidation by superoxide to produce red fluorescent products. Our results demonstrated a significant increase in DHE staining fluorescence intensity in the MOC-Mn group compared to both the control group and MnCl_2_ group, indicating elevated ROS production induced by MOC-Mn (Figure 5D). Additionally, the anti-tumor effect was evaluated through histological analysis of subcutaneous tumor tissues using H&E staining; evident damage was observed in the tumor tissue after treatment with MOC-Mn compared to both control and MnCl_2_-treated groups (Figure 5E).

To assess the potential of MOC-Mn for tumor-specific imaging, T1-weighted MRI was performed on tumor-bearing mice using an MR scanner. As shown in Figure 5F, both control and MnCl_2_-treated groups exhibited relatively low MR signal, whereas the administration of MOC-Mn significantly enhanced the T1-MR contrast in the tumor area. This observation directly indicated the highly sensitive T1-MRI performance of MOC-Mn, which was conducive to advancing integrated diagnosis and treatment.

## Conclusions

In summary, we have successfully engineered an atomically precise theranostic nanoplatform based on metal organic cage with MRI-guided chemodynamic therapy performance for cancer treatment. The MOC-Mn elicited the generation of ROS by means of a Fenton-like reaction and suppressed HeLa cells via mitochondrial depolarization and the subsequent induction of pyroptosis, manifesting considerable potential for anticancer therapy. Furthermore, the T1-weighted MR imaging effect of MOC-Mn enables real-time dynamic monitoring of the anticancer therapeutic process. Additionally, MOC-Mn demonstrated excellent biocompatibility and exhibited potent efficacy in eradicating cancer cells, thus enabling precise cancer therapeutics. Our study advances the development of precise nanoplatforms integrating diagnosis and treatment of diseases, showcasing significant potential for clinical translation.

## Supplementary Material

Supplementary figures and tables.

## Abbreviations

ATR: attenuated total refraction
CDT: chemodynamic therapy
DCFH-DA: 2',7'-dichlorodihydrofluorescein diacetate
DDW: double distilled water
DLS: dynamic light scattering
DMPO: 5,5-dimethyl-1-pyrroline N-oxide
EDS: energy dispersive spectrometer
EPR: electron paramagnetic resonance
FT-IR: flourier transform infrared spectroscopy
H_3_BTC: 1,3,5-benzenetricarboxylic acid
MALDI-TOF: matrix assisted laser desorption/ionization time of flight
MMP: mitochondrial membrane potential
MOC: metal organic cage
MRI: magnetic resonance imaging
MTT: methyl thiazolyl tetrazolium
PDI: polydispersity indices
PXRD: powder X-ray diffraction
RBCs: red blood cells
SC4A: 4-tert-butylsulfonylcalix[4]arene
SC-XRD: single-crystal X-ray diffraction
SEM: scanning electron microscope
TME: tumour microenvironment
WB: western blot
XPS: X-ray photoelectron spectroscopy
