# Supplementary material for: Metal-organic cage as a theranostic nanoplatform for magnetic resonance imaging guided chemodynamic therapy
Source: Theranostics. 2024 Aug 12;14(12):4861–73. doi: 10.7150/thno.97264 (PMC11373615; doi:10.7150/thno.97264)
Supplement: Supplementary file 1 — Supplementary figures and tables. [file thnov14p4861s1.pdf]

Supporting Information

Metal Organic Cage as Theranostic Nanoplatform for MRI Guided Chemodynamic Therapy

Peilin Yin[1,2,4#], Demei Sun[1,2#], Yucen Deng[1,2], Xinyuan Zhu[2], Youfu Wang[2*], Jinghui Yang[3*], and Xuesong Feng[1*]

1. School of Pharmacy, China Medical University, Shenyang 110122, China.

2. School of Chemistry and Chemical Engineering, Shanghai Jiao Tong University, Shanghai 200240, China.

3. Department of Organ Transplantation, Shanghai Changzheng Hospital, Naval Medical University, Shanghai 200003, China.

4. School of Light Industry & Chemical Engineering, Dalian Polytechnic University, Dalian 116034, China

# These authors contributed equally to this work.

Corresponding authors: Youfu Wang (wyfown@sjtu.edu.cn), Jinghui Yang (yjh@smmu.edu.cn), Xuesong Feng (xsfeng@cmu.edu.cn).

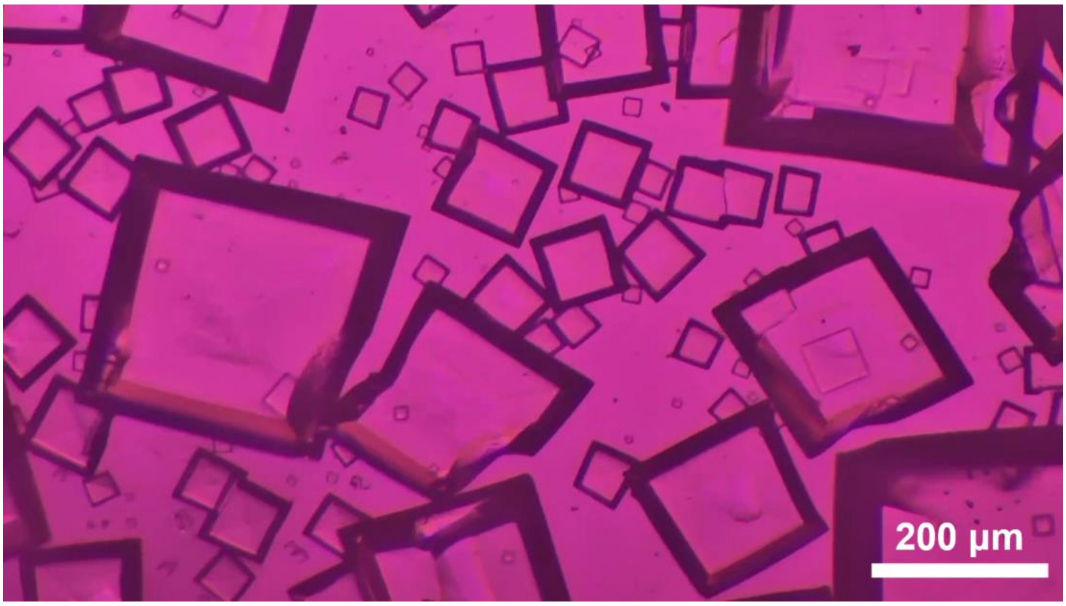

Figure S1. The morphology of crystal MOC-Mn under optical microscope.

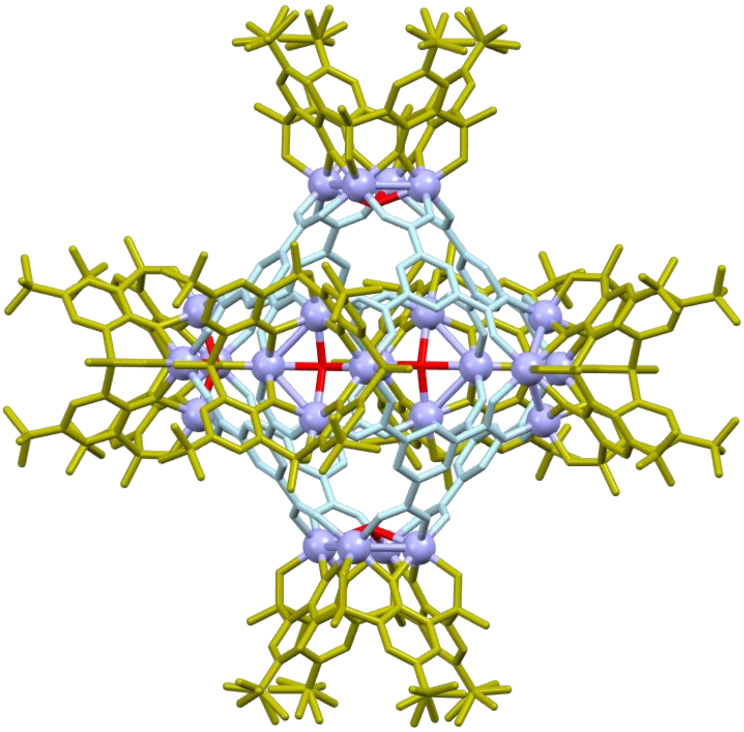

Figure S2. Structure of MOC-Mn from SC-XRD.

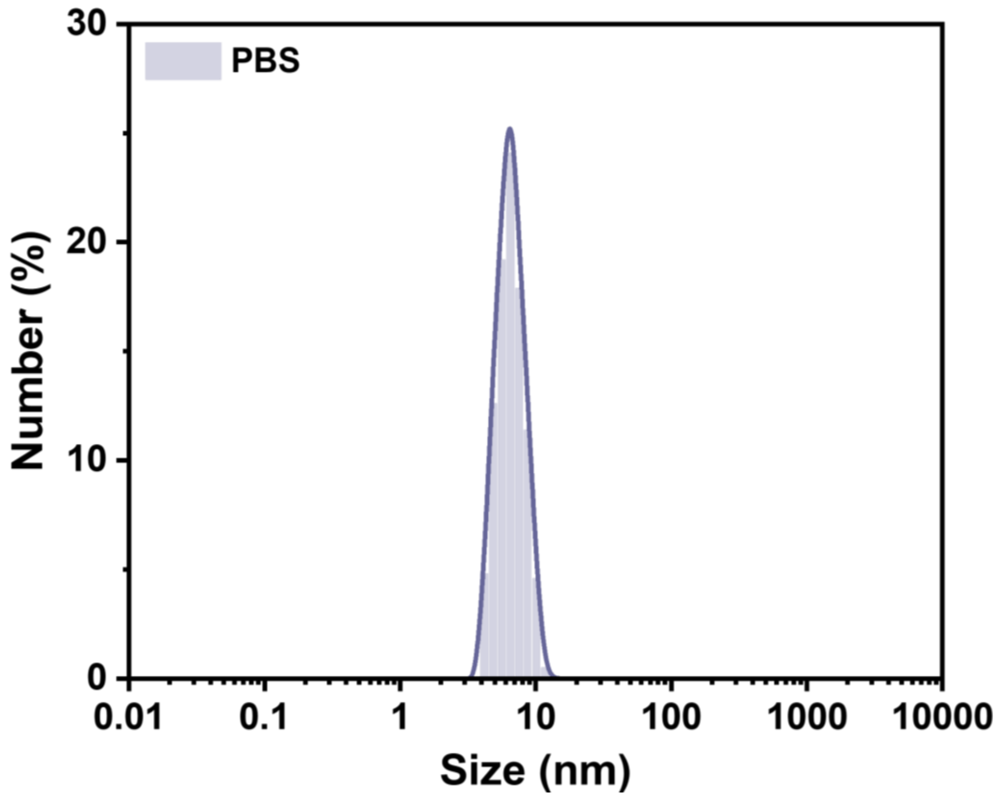

Figure S3. The DLS plots of MOC-Mn in PBS.

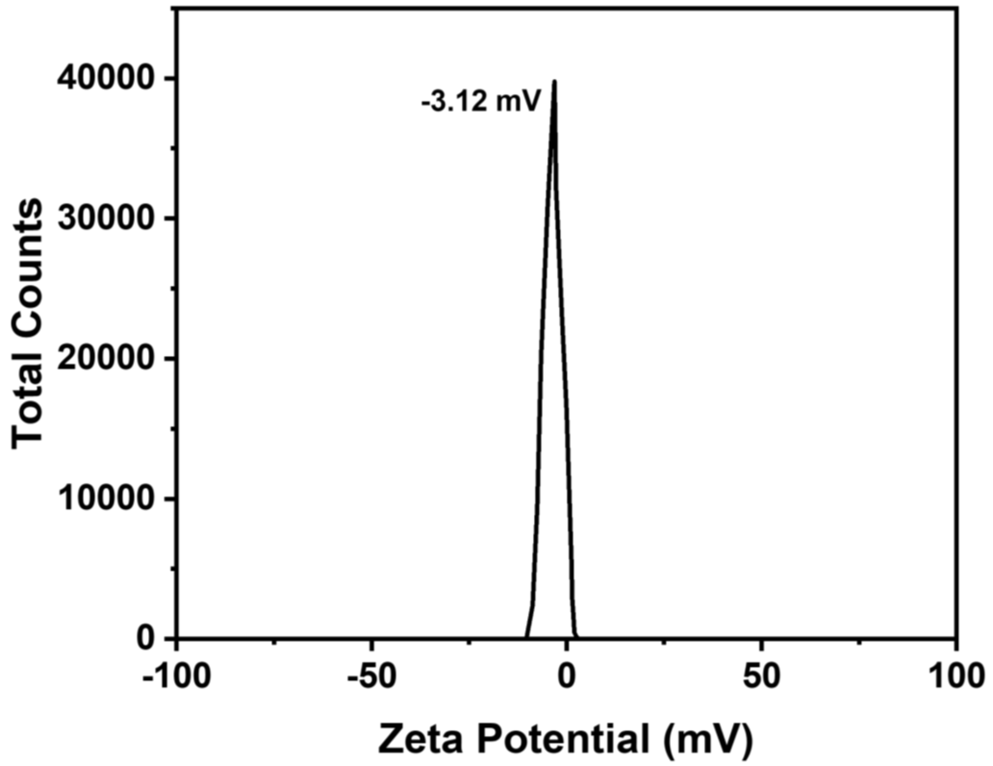

Figure S4. The zeta potential of MOC-Mn in PBS.

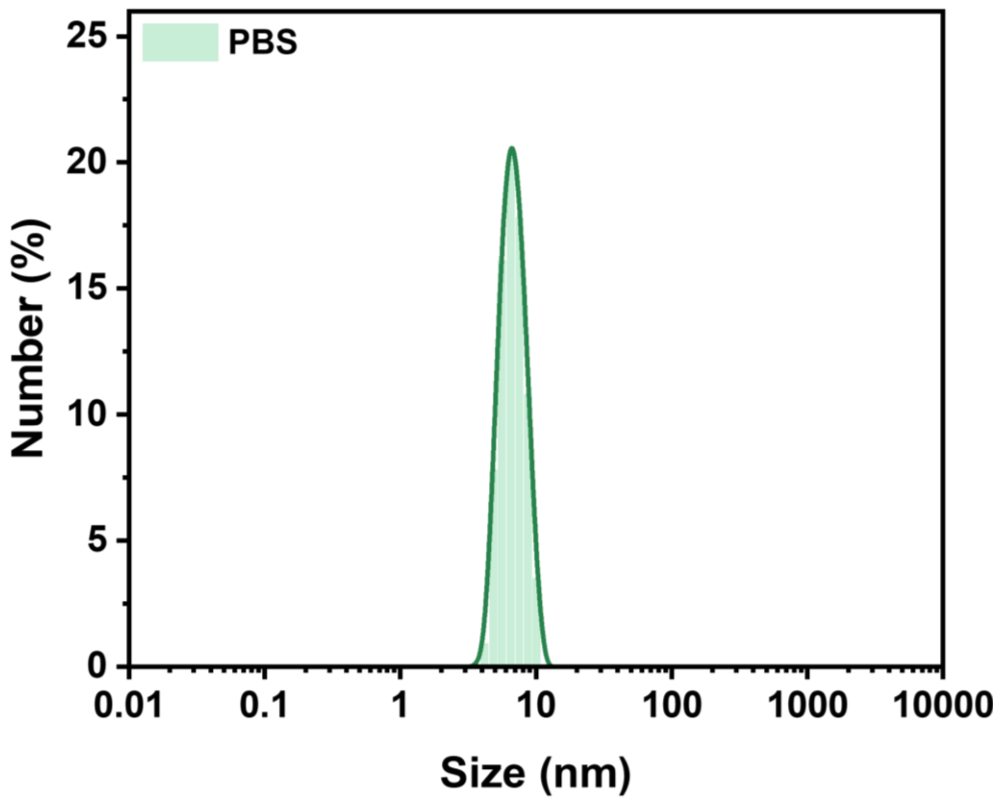

Figure S5. The DLS plots of MOC-Mn in PBS after a 5-day period.

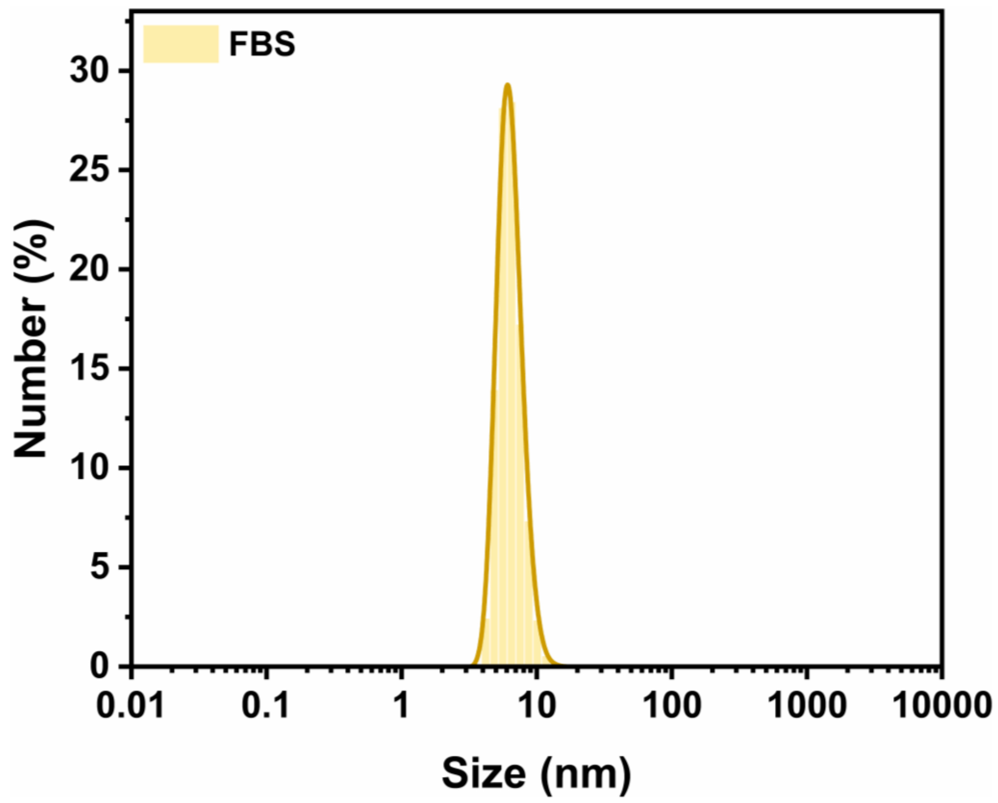

Figure S6. The DLS plots of MOC-Mn in FBS.

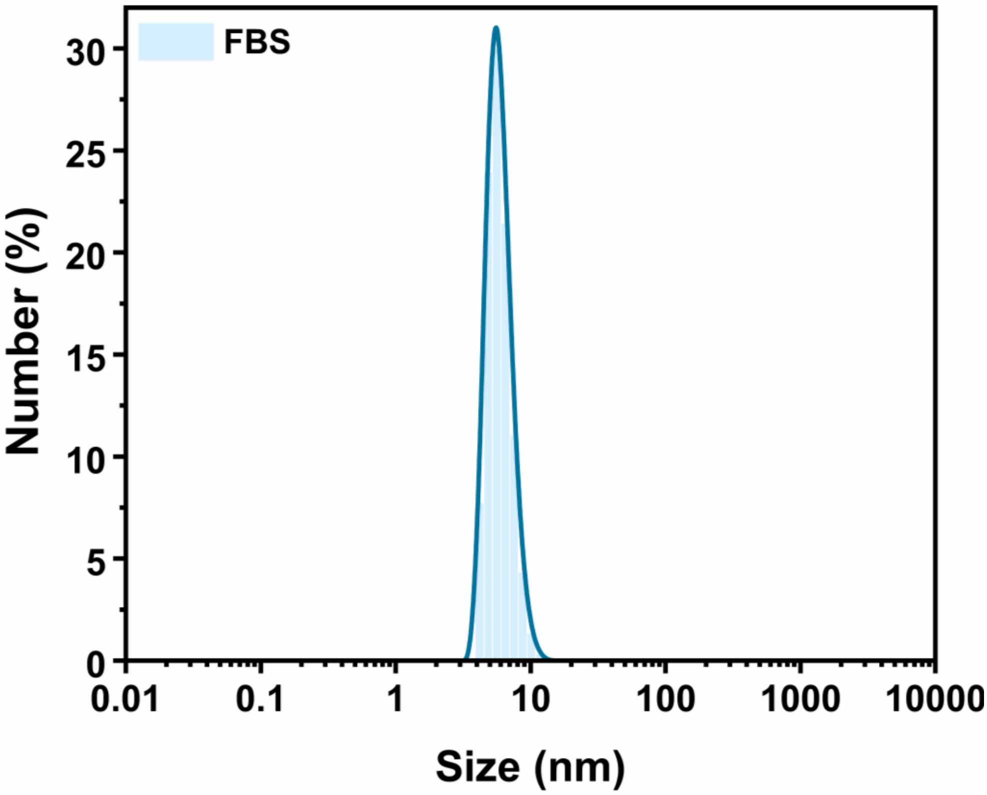

Figure S7. The DLS plots of MOC-Mn in FBS after a 5-day period.

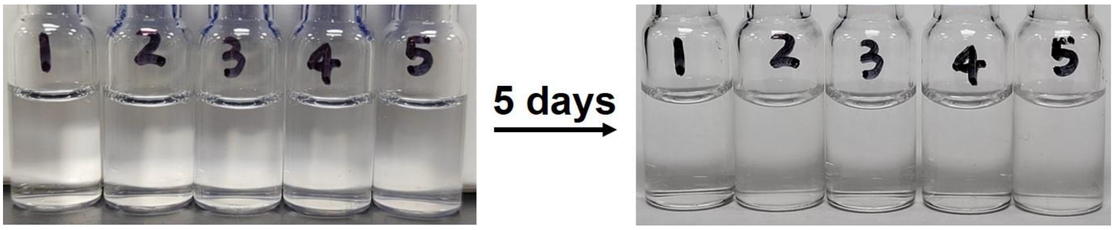

Figure S8. The photos of different concentrations of MOC-Mn (dissolved in DMSO) in PBS after a 5-day period.

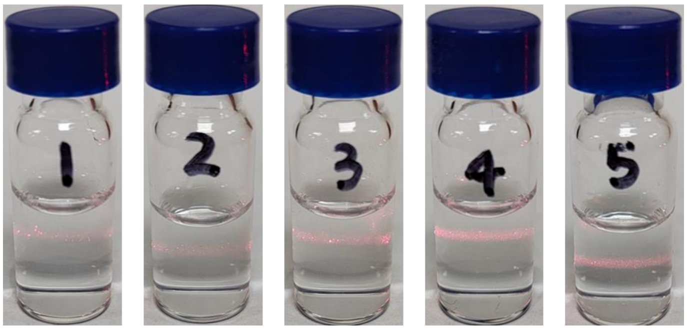

Figure S9. The Tyndall effect of MOC-Mn (dissolved in DMSO) in PBS. The final concentrations are 25, 50, 75, 100, and 125 μg/ml (designated as 1-5 in the picture).

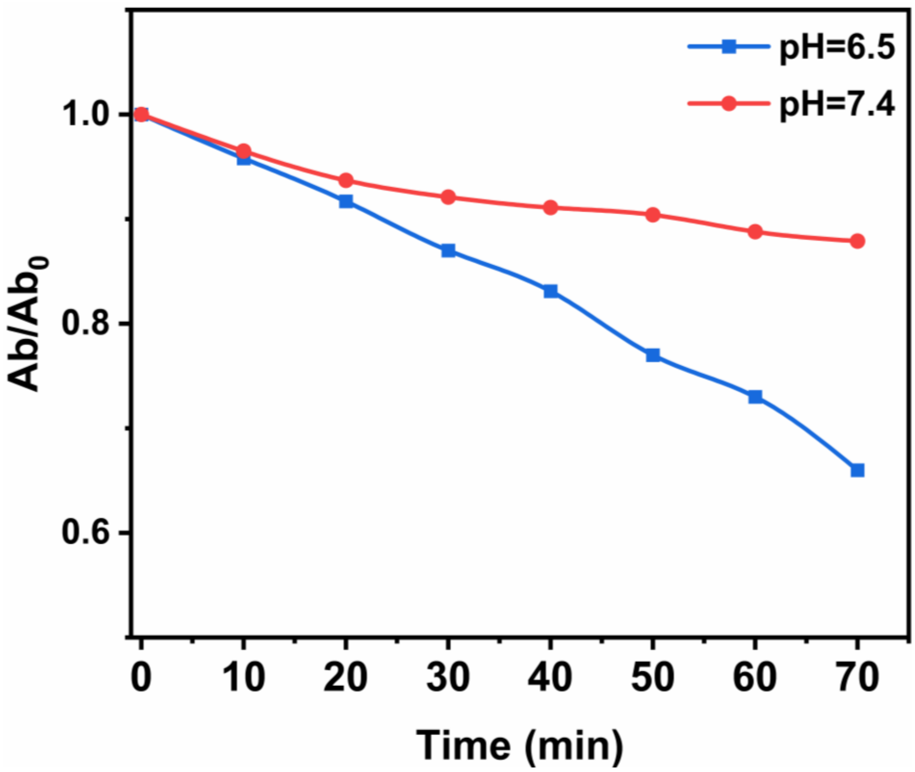

Figure S10. The UV-vis absorption spectra of MB after treatment with MOC-Mn under different conditions.

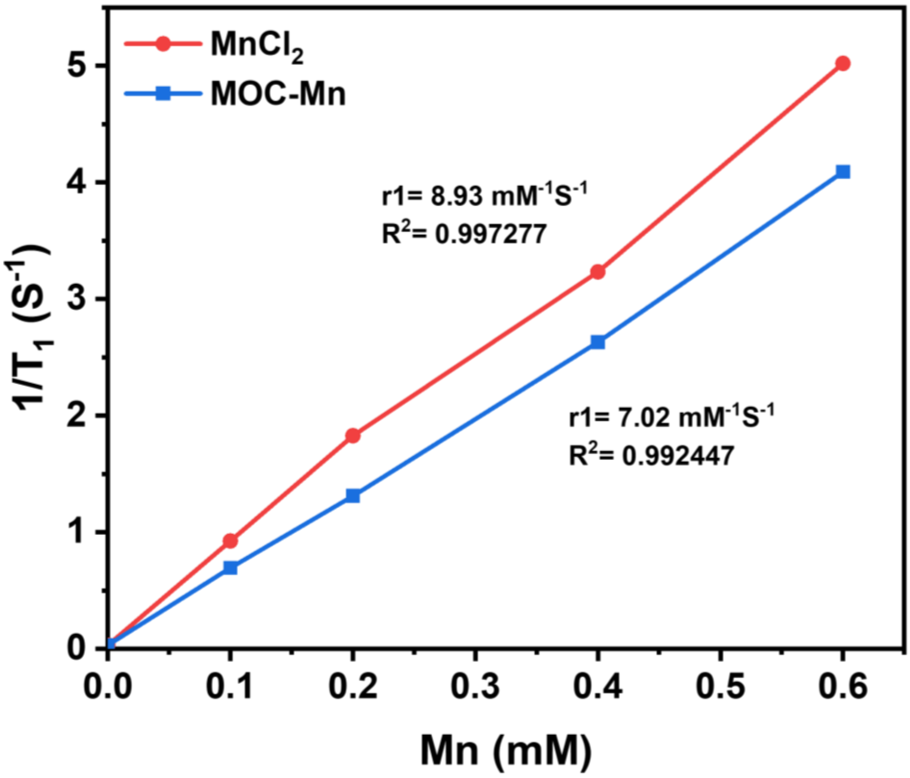

Figure S11. The relaxation rate of $MnCl_2$ and MOC-Mn.

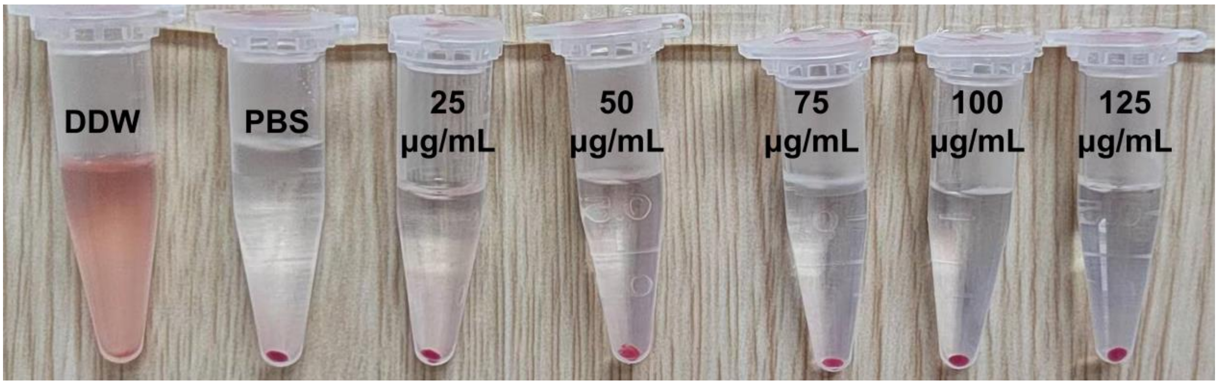

Figure S12. The hemolytic test of MOC-Mn in different concentrations.

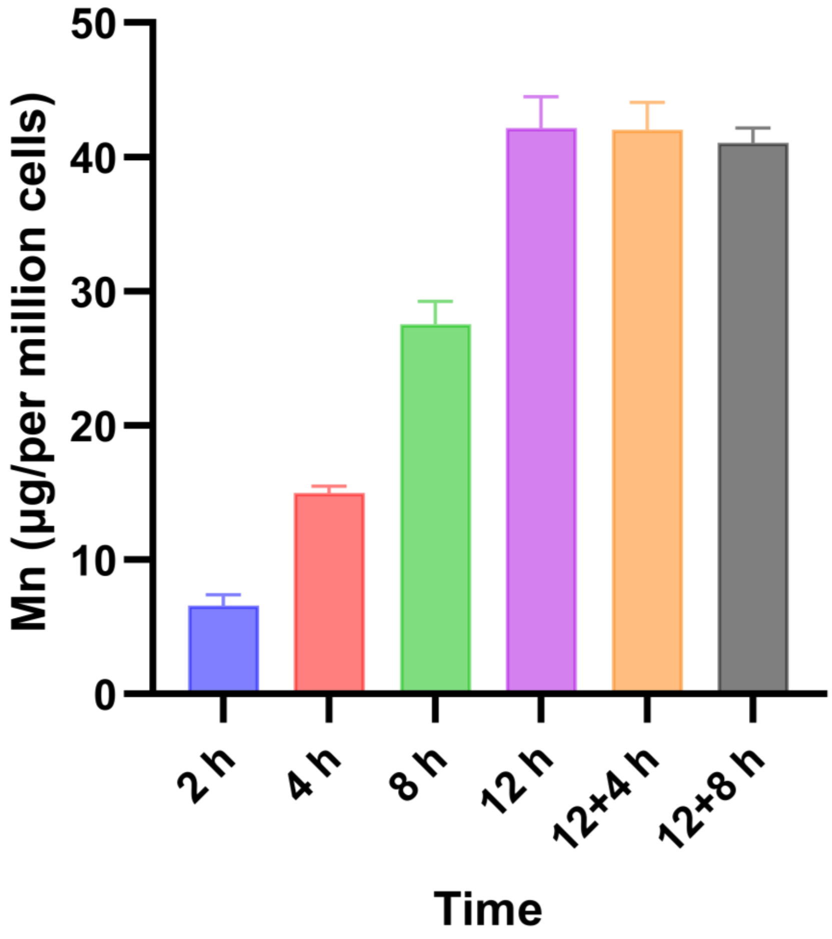

Figure S13. Cellular uptake and retention of MOC-Mn in HeLa cells.

Table S1. Crystal data and structure refinement for MOC-Mn.

Name	MOC-Mn
Empirical formula	$C_{312}H_{288}Mn_{24}O_{126}S_{24}$
Formula weight	8141.41
Temperature/K	100
Crystal system	Tetragonal
Space group	*I*4/m
a/Å	26.6066 (5)
b/Å	26.6066 (5)
c/Å	44.5181 (13)
α/°	90
β/°	90
γ/°	90
Volume/Å^3	31514.9(15)
Z	2
ρ_{calc}g/cm^3	0.883
Mμ/mm^{-1}	4.946
F(000)	8552.0
h,k,l_{max}	32,32,53
Nref	14737
Radiation	Cu Kα (λ=1.54184)

Table. S2 The elemental distribution of MOC-Mn from EDS mapping.

Element	Atomic %	Atomic % Error	Weight %	Weight % Error	Net Counts
C	64.8	0.3	55.7	0.2	364 257
O	33.2	0.3	38.0	0.3	72 524
S	0.8	0.0	1.9	0.0	16 718
Mn	1.1	0.0	4.4	0.1	7 604
